# Association of frailty and physical function in patients with non-dialysis CKD: a systematic review

**DOI:** 10.1186/1471-2369-14-228

**Published:** 2013-10-22

**Authors:** Simon R Walker, Kamalpreet Gill, Kerry Macdonald, Paul Komenda, Claudio Rigatto, Manish M Sood, Clara J Bohm, Leroy J Storsley, Navdeep Tangri

**Affiliations:** 1 Seven Oaks Hospital, Winnipeg, Manitoba, Canada; 2 St Boniface Hospital, Winnipeg, Manitoba, Canada; 3 University of Manitoba, Winnipeg, Manitoba, Canada; 4 Health Sciences Centre, Winnipeg, Manitoba, Canada

**Keywords:** Chronic kidney disease, Elderly, Frailty, Physical function

## Abstract

**Background:**

Frailty is a condition characterized by a decline in physical function and functional capacity. Common symptoms of frailty, such as weakness and exhaustion, are prevalent in patients with chronic kidney disease (CKD). The increased vulnerability of frail patients with coexisting CKD may place them at a heightened risk of encountering additional health complications. The purpose of this systematic review was to explore the link between frailty, CKD and clinical outcomes.

**Methods:**

We searched for cross sectional and prospective studies in the general population and in the CKD population indexed in EMBASE, Pubmed, Web of Science, CINAHL, Cochrane and Ageline examining the association between frailty and CKD and those relating frailty in patients with CKD to clinical outcomes.

**Results:**

We screened 5,066 abstracts and retrieved 108 studies for full text review. We identified 7 studies associating frailty or physical function to CKD. From the 7 studies, we identified only two studies that related frailty in patients with CKD to a clinical outcome. CKD was consistently associated with increasing frailty or reduced physical function [odds ratios (OR) 1.30 to 3.12]. In patients with CKD, frailty was associated with a greater than two-fold higher risk of dialysis and/or death [OR from 2.0 to 5.88].

**Conclusions:**

CKD is associated with a higher risk of frailty or diminished physical function. Furthermore, the presence of frailty in patients with CKD may lead to a higher risk of mortality. Further research must be conducted to understand the mechanisms of frailty in CKD and to confirm its association with clinical outcomes.

## Background

Chronic kidney disease (CKD) is a major public health problem affecting an estimated 1.2 million Canadians [1]. Although the overall prevalence of CKD is roughly 10% in the general population, the disease increases with age, affecting more than one third of all individuals over age 65 [2]. In this population, CKD is associated with additional comorbid conditions; a higher risk of cardiovascular disease and increasing prevalence of frailty and disability [3,4].

Frailty is a multidimensional syndrome characterized by loss of lean body mass (sarcopenia), weakness and decreased endurance, leading to reduced activity and a poor response to stressors [5]. Reduced activity in turn worsens sarcopenia and weakness, causing a downward functional spiral and increasing risk of death. This is often exacerbated by social isolation and depression that reinforces behaviors leading to further inactivity, disuse and loss of function [6]. Indeed, several prospective studies have shown that measures of frailty are strongly associated with death and hospitalization in older individuals and moreover that this association is independent of other clinical risk factors and comorbid conditions [7].

The interrelationship between frailty, chronic diseases and adverse outcomes in older people has been recognized as an important focus of research in patients with cardiovascular disease (CVD) and in patients on dialysis [8,9]. However, studies examining frailty in non-dialysis CKD are limited. This is a significant omission given that CKD correlates with increasing age thereby afflicting a similar demographic as frailty [5]. Many of the physiological changes resulting from CKD are altered mineral metabolism, chronic inflammation and arteriosclerosis. These physiological changes directly or indirectly influence sarcopenia and weakness, which are core domains of the frailty construct [10]. Conversely, frailty may adversely affect adaptations to the numerous health state transitions that CKD patients undergo over time. As such, individuals with coexisting frailty and CKD may be at a synergistic risk of experiencing adverse clinical outcomes. Given that frailty may be modifiable using readily available interventions, the study of frailty in the patient with CKD seems particularly important [11].

In order to identify the gaps in evidence in this population, we performed a systematic review to identify all relevant studies examining the association between frailty, physical function and CKD. We hypothesized that patients with CKD are more likely to be characterized as frail or functionally limited, and that the coexistence of the two conditions confers a higher likelihood of adverse clinical outcomes.

## Methods

### Search strategy and selection criteria

We aimed to identify observational studies of humans that explored the link between frailty, CKD, and clinical outcomes. The primary question of interest was the association between frailty and CKD and the likelihood of adverse clinical outcomes in frail subjects with CKD not currently on dialysis therapy. The selection criterion was broadened to include studies that looked at individual domains of frailty, such as functional limitation.

### Data sources and search strategy

In collaboration with a medical librarian (K.M.), a search strategy was designed and implemented to capture all relevant studies from the available literature. The following electronic databases were searched from their date of establishment to March 2013: EMBASE, PubMed, Web of Science (WOS), CINAHL, Cochrane Database of Systematic Reviews (CDSR), Database of Abstracts of Reviews of Effects (DARE), Cochrane Central Register of Controlled Trials (CENTRAL), and AgeLine. The search strategy was tailored to each database and used a combination of key words such as “frailty,” “elderly,” and “kidney disease,” as well as MeSH terms (see Additional file 1: Appendix 1).

### Article eligibility criteria

The eligibility for full text review of each citation was independently evaluated by two reviewers (S.W. and K.G.) on the basis of title and abstract. Any article that was deemed potentially relevant by either reviewer was retrieved for full-text review. The reference lists of any relevant review articles were also screened to identify studies which may have potentially been missed in the search. The reviewers then independently assessed the full text articles and studies were finalized for inclusion in the systematic review after discussion with a third reviewer (N.T.). Disagreements were resolved by consensus.

### Data extraction

A data extraction form was created to capture relevant information from included studies. One reviewer (S.W.) independently conducted the extraction with verification by a second reviewer (K.G.). The following information was extracted for each study: 1. study characteristics, such as the year of publication, study design, study population, and sample size; 2. definitions of CKD and frailty (or its associated domains); and 3. patient characteristics, such as mean age, percentage of males, race and comorbidities.

### Evaluation of risk of bias

We evaluated each study for risk of bias using the Newcastle-Ottawa Scale (NOS) [12] in order to assess its quality. The NOS is a quality evaluation method for non-randomized studies. The NOS criteria are split into three sections: Selection, Comparability and Outcome. Each study is designated a number of stars for each section, based on predetermined queries. The queries used in this systematic review can be found in Additional file 1: Appendix 2.

### Statistical analysis

As significant clinical heterogeneity in the patient population, definitions of frailty, study methodologies and covariate adjustment were encountered; meta-analysis was not performed. In order to assess publication bias, we created funnel plots of the risk associations (Additional file 1: Appendix 3). Cross-sectional and Cohort studies were plotted separately.

### Declaration of sources of funding

No specific funding.

## Results

### Search results and study selection

**Figure 1:**
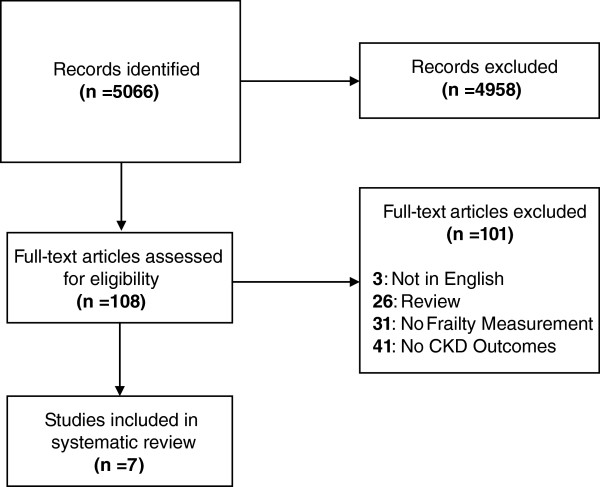
Flow Diagram of Reviewed Studies.

The search retrieved 5,066 individual citations for screening. After conducting the initial screen of these citations, 108 articles were selected for full-text review and of these, 7 articles met criteria for inclusion in the study. The basis of exclusion for the remaining articles is shown in Figure 1[13].

### Study characteristics

The 7 included studies encompassed a total of 20,332 patients. All 7 studies examined the relationship between CKD and frailty (or one its domains), but only two studies related frailty in CKD patients to a clinical outcome [14,15]. The studies were mainly conducted in the United States and published after 2004 with the exception of one conducted in Italy, published in 2012 [16]. Two of the studies were prospective [4,15] and the remaining studies were secondary analyses [14,16-19]. All of the studies provided a measure of kidney function and a description of frailty or one of its domains. Table 1 shows the individual study characteristics for the included studies.

**Table 1:** Characteristics of included studies

**First author, year**	**Population**	**n**^**a**^	**Association**	**Physical measurement**	**Observer or self**-**reporting**	**Kidney function endpoint**	**Endpoint definition**
**CKD and frailty associated with outcome**							
Wilhelm-Leen [14]	age ≥20 3–5 frailty domains measured NHANES III	10,256	Frailty and CKD linked to mortality	Modified Fried Frailty Criteria	Both	**Chronic Kidney Disease** Stage 5	CKD Stage 5 definition: eGFR <45 mL /min/1.73 m^2^
Roshanravan [15]	age ≥18 CKD Stages 1–4 not requiring dialysis	336	Frailty associated with lower eGFR and higher risk of death or dialysis therapy	Modified Fried Frailty Criteria	Both	**Chronic Kidney Disease**	CKD definition: eGFR <90 mL /min/1.73 m^2^ OR albuminuria^b^
**CKD and frailty associated**							
Odden [17]	previous cardiac event or disease	954	CKD associated with physical limitation and reduced exercise capacity	Exercise and Functional Capacity	Both	**Chronic Kidney Disease** Stage 3 or higher	CKD Stage 3 definition: Creatinine clearance <60 ml/min
Shlipak [18]	Medicare eligibility age > 65 non-institutionalized	5,808	CRI associated with frailty in community dwelling elder adults	Fried Frailty Criteria	Both	**Chronic Renal Insufficiency**	CRI definition: Male = serum creatinine >1.5 mg/dL, Female = serum creatinine >1.3 mg/dL
Fried [4]	Medicare eligibility age 70–79 no existing functional limitation	2,135	CKD associated with development of impaired physical function	Functional Limitation	Self	**Kidney Function** Quartile	Cystatin C input to MDRD. Secondary analysis grouped patients as eGFR <60 mL /min/1.73 m^2^ vs other
Bowling [19]	Community-dwelling Medicare beneficiaries age ≥65	357	CKD associated with functional decline	IADLs and BADLs	Self	**Chronic Kidney Disease** Stage 3 or higher	CKD definition: eGFR <60 mL /min/1.73 m^2^
Lattanzio [16]	Older Hospitalized Patients	486	Reduced kidney function associated with poor physical performance	Short Physical Performance Battery	Observer	**Chronic Kidney Disease** Stage 3 or higher	CKD definition: eGFR <60 mL /min/1.73 m^2^

*BADL* Basic activities of daily living, *CKD* Chronic Kidney Disease, *CRI* Chronic Renal Insufficiency, *DM* diabetes mellitus, *eGFR* estimated glomerular filtration rate, *IADL* Instrumental activities of daily living, *MDRD* Modification of Diet in Renal Disease equation, *NHANES* III, third National Health and Nutrition Evaluation Survey, *RRT* renal replacement therapy.

^a^study sample size.

^b^Albuminuria defined as urine albumin-creatinine ratio >30 mg/g from a 12-hour urine collection.

### Measures of frailty and physical function

Each study used different methods for the definition and identification of frailty and physical function (Table 2). Both of the studies examining the association between frailty and adverse clinical outcomes relied on a modified version of the Fried frailty criteria. Wilhelm-Leen et al. abbreviated multiple measures and relied on single item questions to define the weakness, exhaustion and physical activity domains of the frailty definition. In contrast, Roshanravan et al. used objective criteria for weakness, but defined exhaustion from the results of a survey (SF-36) and slowness from the results of a 4 meter walk test.

**Table 2:** Frailty and physical function criteria

**First author, year**	**Physical measurement**	**Physical measurement method**	**Outcome**	**Criteria**
**CKD and frailty associated with outcome**				
Wilhelm-Leen [14]	*Modified Fried Frailty Criteria*	• Low body weight (BMI ≤ 18.5 kg/m^2^)	Frailty	3 or more frailty domains present
		• Slow walking speed in timed 8 foot walk (lowest 20% adjusted for gender)		
		• Self reported weakness		
		• Self reported exhaustion		
		• Self reported low physical activity		
Roshanravan [15]	*Modified Fried Frailty Criteria*	• Unintentional weight loss (≥10 lbs in last 6 months)	Frailty or Intermediate Frailty	Frailty: 3 or more frailty domains Intermediate Frailty: 1 or 2 frailty domains present
		• Grip Strength (lowest 20% adjusted for gender and BMI)		
		• Self reported Exhaustion (lowest 20% score on SF-36)		
		• Slow walking speed in timed 15 foot walk (lowest 20% adjusted for gender and height)		
		• Self reported low physical activity (exercise <1 per week)		
**CKD and frailty associated**				
Odden [17]	*Exercise and Functional Capacity*	• Self-perception of physical function (assessed by SAQ)^a^	Physical Limitation and/or low Exercise Capacity	Physical Limitation: SAQ score <75
		• Objective exercise capacity (treadmill test evaluated by Bruce protocol)^b^		Low Exercise Capacity: maximum exercise capacity <5 MET
Shlipak [18]	*Fried Frailty Criteria:*	• Unintentional weight loss (≥ 10 lbs or ≥ 5% loss in body weight)	Frailty	3 or more frailty domains present
		• Slow walking speed on 15 foot walk (lowest 20% adjusted for gender and height)		
		• Grip Strength (lowest 20% adjusted for gender and BMI)		
		• Poor endurance and energy (CES-D questionnaire)		
		• Self reported low physical activity (lowest 20% adjusted for gender)		
Fried [4]	*Physical Function*	• Self-reported difficulty in climbing 10 steps or walking one quarter of a mile	Functional Limitation	Two consecutive reports of difficulty involving the same function (stairs or walking, measured every 6 months)
Bowling [19]	*Functional Decline*	• IADL Score^c^	Functional Limitation	IADL or BADL score lower than baseline values
		• BADL Score^d^		
Lattanzio [16]	*Short Physical Performance Battery*	• Slow walking speed test (scored 0–4)	Good, Intermediate or Poor physical performance	Grouping by SPPB Score: 0–4, 5–8 and 9-12
		• Chair stand test(scored 0–4)		
		• Balance test (scored 0–4)		

*BADL* Basic Activities of Daily Living, *BMI* body mass index, *CES*-*D* Center for Epidemiologic Studies Depression Scale, *IADL* Instrumental activities of daily living, *MET* metabolic equivalent tasks *SPPB*, Short Physical Performance Battery, *SAQ* Seattle Angina Questionnaire. SF-36, 36-Item Short Form Health Survey.

^a^SAQ scored from 0-100, High Score denotes better physical function.

^b^Bruce Protocol: maximum exercise capacity determined by total number of MET achieved in test.

^c^IADLs list: using the telephone, light housework, heavy housework, preparing meals, shopping and managing money.

^d^BADL list: bathing, transferring out of a bed or chair, eating, toileting and dressing.

The studies examining an association between frailty or physical function and CKD used many different definitions of decreased physical function. One study [18] used unmodified Fried frailty criteria. Two studies used standardized measurements to identify reduced physical ability, measuring exercise and functional capacity [17] and completing a Short Physical Performance Battery [16] test. Two studies allowed patients to self-report whether they were functionally limited [4] or whether they had difficulty with activities of daily living [19]. A detailed description of the frailty/physical function criteria in each study is listed in Table 2.

### Association of frailty with CKD

We found 7 studies that examined the association of frailty or its individual components with CKD. The largest study in our analysis utilized data from the third National Health and Nutrition Evaluation Survey (NHANES III) and examined the association between frailty as defined by the modified Fried criteria and CKD defined by serum creatinine based eGFR [14]. The results suggested that patients in the early stages of CKD were at a greater than two-fold higher risk of being frail in comparison to healthy patients [CKD stage 1/2: odds ratio (OR) 2.21, 95% confidence interval (CI) 1.49-3.28; CKD stage 3a: OR 2.48, 95% CI 1.57-3.93]. Furthermore, patients with CKD at or beyond stage 3b were found to be nearly six times as likely to be frail (OR 5.88, 95% CI 3.40-10.16). A similar community based study, using the Cardiovascular Health Study population, also found consistent results, but the association was weaker (OR 1.76, 95% CI 1.28-2.41 [18]. Finally, a more recent study [15] , in the advanced CKD population, also showed similar findings. In this population, patients with and eGFR in the range of 30–44 mL/min/1.73 m^2^ were two times more likely to have the frailty phenotype (OR 2.1, 95% CI 1.0-4.7) and patients with an eGFR measurement of less than 30 mL/min/1.73 m^2^ were nearly three times more likely to be frail (OR 2.8, 95% CI 1.3-6.3).

The remaining four studies largely focused on the physical function domains of frailty such as functional limitation, exercise capacity and physical performance in relation to CKD. One of the four studies used creatinine clearance as a marker of kidney function. The remaining three studies utilized eGFR measurements to determine kidney function. In these four studies, CKD was consistently associated with a higher risk of developing a limitation in the physical function domain of frailty that each respective study examined. A summary of the associations between CKD, frailty and/or frailty domains reported by each of the studies is seen in Table 3.

**Table 3:** Associations reported within included studies

**First author, year**	**Association**	**Analysis**
**CKD and frailty associated with outcome**		
Wilhelm-Leen [14]	• Mortality in frail patients with CKD	HR 2.0, 95% CI 1.5 - 2.7
	• Frailty in patients with CKD stage 1/2	OR 2.21, 95% CI 1.49-3.28
	• Frailty in patients with CKD stage 3a	OR 2.48, 95% CI 1.57-3.93
	• Frailty in patients with CKD stage 3b-5	OR 5.88, 95% CI 3.40-10.16
Roshanravan [15]	• Frailty components associated with Death or Dialysis	HR 2.5, 95% CI 0.9-2.87
	• Frailty with eGFR between 30- 44 mL/min/1.73 m^2^	HR 2.1, 95% CI 1.0-4.7
	• Frailty with eGFR <30 mL/min/1.73 m^2^	HR 2.8, 95% CI 1.3-6.3
**CKD and frailty associated**		
Odden [17]	• Physical limitation with creatinine clearance between 60-90 mL/min	OR 1.0, 95% CI 0.7-1.5
	• Physical limitation with creatinine clearance <60 mL/min	OR 2.0, 95% CI 1.3-3.1
	• Low exercise capacity with creatinine clearance between 60-90 mL/min	OR 2.3, 95% CI 1.4-3.8
	• Low exercise capacity with creatinine clearance <60 mL/min	OR 6.7, 95% CI 3.8-11.8
Shlipak [18]	• Frailty in patients with CRI	OR 1.76, 95% CI 1.28-2.41
Fried [4]	• Functional limitation in patients with CKD (eGFR <60 mL/min/1.73 m^2^)	HR 1.30, 95% CI 1.08-1.56
Bowling [19]	• IADL decline with CKD stage≥3B (eGFR <45 mL/min/1.73 m^2^)	OR 3.12, 95% CI 1.38-7.06
	• BADL decline with CKD stage≥3B (eGFR <45 mL/min/1.73 m^2^)	OR 3.78, 95% CI 1.36-9.77
Lattanzio [16]	• Low SPPB total score and eGFR	B = 0.49, 95% CI 0.18-0.66, *p*=0.003

*BADL* Basic Activities of Daily Living, *CI* confidence interval, *CKD* Chronic Kidney Disease, *CRI* chronic renal insufficiency, *HR* hazard ratio, *IADL* Instrumental activities of daily living, *OR* odds ratio, *SPPB* Short Physical Performance Battery.

### Association of frailty with clinical outcomes in patients with CKD

Only two published studies related frailty in patients with CKD to an adverse clinical outcome [14,15]. One study used data from NHANES III and related frailty in patients with CKD to mortality. After adjusting for multiple comorbid conditions, frailty in CKD patients was associated with a two-fold higher risk of mortality compared to CKD patients who were not frail [hazard ratio (HR) 2.0, 95% CI 1.5-2.7]. The second study was performed in a smaller population with more advanced CKD and used Death and Dialysis as the clinical outcomes. The study adjusted for age, sex, BMI, eGFR, diabetes and cardiovascular disease and found that the frailty phenotype was associated with a 2.5 fold greater risk of death or dialysis therapy (HR 2.5, 95% CI 0.9-2.87).

### Risk of bias

The two studies associating Frailty and CKD with clinical outcomes had a similar risk of bias. Both studies has relatively low risk of bias in selection and comparability domains, but had a higher risk of bias in evaluation of outcome. The remaining five studies associated frailty or physical function and CKD and all had a similar risk of bias. A detailed description of the quality assessment is described in Table 4.

**Table 4:** **Newcastle**-**Ottawa scale quality evaluation**

**First author, year**	**Selection**	**Comparability**	**Outcome**	**Total**
**CKD and frailty associated with outcome**				
Wilhelm-Leen [14]	3	2	0	5
Roshanravan [15]	2	2	1	5
**CKD and frailty associated**				
Odden [17]	3	2	0	5
Shlipak [18]	3	2	0	5
Fried [4]	4	2	1	7
Bowling [19]	4	1	1	6
Lattanzio [16]	3	2	0	5

Newcastle-Ottawa Scale Quality Criteria can be found in Additional file 1: Appendix 2.

## Discussion

In our systematic review, we found multiple studies confirming independent associations between frailty, diminished physical function and CKD. There was an association between presence of CKD and being frail or having limitations of physical function. More importantly, in patients with CKD, the presence of frailty was associated with all-cause mortality and dialysis after adjusting for multiple comorbid conditions. Our findings highlight the importance of frailty as both a health outcome and an independent predictor of adverse outcomes in CKD. This has important implications for the clinical care of these patients and for the design of epidemiological studies in this population. In addition, our review also confirms the paucity of literature examining the relationships between frailty, kidney function and clinical outcomes.

To our knowledge, our systematic review is the first to explore frailty and physical function in the CKD population. A previous systematic review explored the association between frailty and CVD [8]. The study showed that a possible bidirectional relationship exists between frailty and CVD and found that frailty in the CVD population was a powerful predictor of mortality. The CVD review was different from ours in that the investigators adhered to strictly defined frailty criteria when choosing studies to be included in their review, rather than a more open-ended definition of frailty and physical function in our study. In the kidney failure population, a single prospective study exploring frailty amongst patients with end-stage renal disease (ESRD) analyzed data collected in the Dialysis Morbidity and Mortality Study (DMMS) Wave 2 [9]. Their definition of frailty was similar to the modified Fried frailty because it incorporated poor self-reported physical functioning, exhaustion/fatigue, low physical activity and under-nutrition. They discovered that nearly two thirds of the examined cohort of dialysis patients met their definition of frailty. Furthermore, they found that frailty in patients on dialysis was independently associated with mortality. In addition, the investigators conducting this analysis were also able to examine components of frailty individually and found that each component was independently associated with mortality in patients with kidney failure.

Two of the seven studies included in our review examined the association between frailty and clinical outcomes in patients with CKD, and also studied the association between prevalent CKD and frailty [14,15]. Both studies defined frailty using modified Fried criteria and utilized self-reported criteria items to define the exhaustion and physical activity domains of frailty. Only one of the two studies followed the Fried criteria for weight loss and weakness, the other defined weight loss as a cross sectional BMI <18.5 and used self-reporting for weakness measurements [14]. The lack of standardized definitions for each of the domains, as well as retrospective adaptation of questionnaires to fit the frailty domains, limits comparisons between these studies. In fact, recent studies have shown that defining frailty based on self-reported questionnaires may lead to significantly higher perceived prevalence than using measured constructs, and therefore further study of frailty using more objective measures is needed [20].

Of the remaining five studies in our review, only one study utilized the Fried frailty criteria. This study [18] only used self-reported values for exhaustion and low physical activity measurements. The additional four studies examined domains of physical ability to study the association between physical function and CKD. Two of these studies used self-reported values to assess ability to complete activities of daily living [19] and functional limitation [4] as outcomes. The remaining two studies used objective measurements to assess physical ability as exercise and functional capacity [17] or Short Physical Performance Battery tests [16]. None of the studies in our review examined alternative definitions of frailty and most did not assess potentially important domains, including mood and cognition. Alternative domains may be particularly important in patients with CKD, as both cognitive impairment and depressed mood can strongly influence decision making about dialysis modalities for both the patient and the provider. Multiple studies in the kidney failure population have suggested that cognitive impairment and depression are exceedingly common in patients with CKD and we believe that future work on frailty in this population should include study of these potentially modifiable domains [21,22].

There are several direct clinical and research implications to our findings. First, the majority of the studies in our review used questionnaires and physical function tests that can be easily administered in the inter-professional CKD clinic setting. If frailty is indeed confirmed to be independently associated with adverse outcomes in CKD, these assessments could be performed routinely in the renal clinic setting without adding significant resources or patient burden. In addition, multiple studies have shown that simple nutritional and physical function interventions such as nutrition and exercise programs targeted at reducing frailty can have an impact on important functional outcomes in the general population and in patients with kidney failure [23-25]. Given that frailty is a modifiable risk factor in the older general population and the high prevalence of older patients in CKD clinics, studies examining the effects of treating frailty in older patients with CKD are urgently needed.

There are several strengths of our review. Our search strategy included multiple electronic databases in an attempt to ensure that all of the published literature examining frailty and CKD was captured. We manually searched the bibliographies of the included articles to ensure that we achieved a high sensitivity of our search strategy. We also examined the quality of each included study using validated criteria and highlighted deficiencies in evidence and reporting. Finally, we broadened our search criteria to include studies examining any definition of frailty and also studies looking at physical function and functional limitation affording the ability to assess the impact of CKD on individual domains of frailty.

Our study has some limitations. Our review was focused on the published literature and it is possible that publication bias may have played a role and excluded studies showing a lack of association between frailty and CKD. Although, our funnel plots (Additional file 1: Appendix 3) did not show any obvious evidence of publication bias, it is possible that residual bias remains in our study selection. Secondly, most of the studies included in our review were secondary data analyses as opposed to primary prospective studies of frailty in CKD. As such, these analyses came from studies not directly designed to explore the link between frailty and CKD, but instead, had data elements which allowed secondary analyses. Thirdly, the methods for assessing physical function, physical performance and frailty in the studies included in our review were heterogeneous. Conclusions about the relationship between CKD and frailty and/or physical function may be limited by the lack of comparisons between different methods in the same population. Finally, we did not include studies examining frailty in patients undergoing dialysis or kidney transplant. Although frailty has been studied more extensively in the kidney failure population, the physiological alterations and morbidity associated with dialysis treatments may not be applicable to patients with CKD not on dialysis.

## Conclusion

In conclusion, our systematic review confirms the independent association between frailty, CKD and adverse clinical outcomes. Frailty appears to be independently associated with prevalent CKD, and the presence of frailty in CKD may be strongly associated with all-cause mortality. In addition, our review highlights the paucity of literature examining the association between CKD and frailty. Further studies examining the epidemiology of frailty in patients with CKD using objective physical and cognitive function criteria are needed, both to elucidate the underlying mechanisms and to inform clinical trials using targeted interventions.

## Competing interests

The authors declare that they have no competing interests.

## Authors’ contributions

SW was involved with abstract and full text screening, extraction of data from selected articles, drafting of the manuscript and approval of final version of the manuscript. KG was involved with abstract and full text screening, extraction of data from selected articles and drafting of the manuscript. KM was responsible for creating and applying the search strategy and retrieving selected articles. PK was involved in study design and approval of the manuscript. CR was involved in study design and approval of the manuscript. MS was involved in study design and approval of the manuscript. CB was involved in study design and approval of the manuscript. LS was involved in study design and approval of the manuscript. NT conceived of the study, and participated in its design and coordination, helped to draft the manuscript and approved the final version of the manuscript. All authors read and approved the final manuscript.

## Pre-publication history

The pre-publication history for this paper can be accessed here:

http://www.biomedcentral.com/1471-2369/14/228/prepub

## Supplementary Material

Additional file 1 **Appendix 1**: Search Strategy. **Appendix 2**: NOS Criteria. **Appendix 3**: Publication Bias Funnel Plot. Click here for file
