# Supplementary material for: Association of frailty and physical function in patients with non-dialysis CKD: a systematic review
Source: BMC Nephrol. 2013 Oct 22;14:228. doi: 10.1186/1471-2369-14-228 (PMC4016413; doi:10.1186/1471-2369-14-228)
Supplement: Additional file 1 — Appendix 1: Search Strategy. Appendix 2: NOS Criteria. Appendix 3: Publication Bias Funnel Plot. [file 1471-2369-14-228-S1.docx]

Additional file 1: Appendix 1: Search Strategy

(("geriatric assessment"[mesh] OR "age factors"[mesh:noexp] OR "elderly"[All Fields] OR "aged, 80 and over"[mesh] OR "aged"[mesh] OR "frailty"[All Fields] OR "frail"[All Fields] OR "frail elderly"[mesh]) AND (“walking”[mesh] OR “mobility limitation”[mesh] OR “geriatric assessment”[mesh] OR "health services for the aged"[mesh] OR "quality of life"[mesh] OR "fatigue"[mesh] OR "fatigue"[all fields] OR "muscle weakness"[all fields] OR "activities of daily living"[mesh] OR "Patient Selection"[MeSH]) AND ("Kidney"[Mesh] OR "Renal Insufficiency"[Mesh:noexp] OR "Kidney Failure, Chronic"[Mesh] OR "Renal Insufficiency, Chronic"[Mesh:noexp] OR "kidney disease"[tiab])) OR (((“frail elderly" AND “health status”[mesh]) OR (“frail elderly”[mesh])) AND (“Kidney"[Mesh] OR "Renal Insufficiency"[Mesh:noexp] OR "Kidney Failure, Chronic"[Mesh] OR "Renal Insufficiency, Chronic"[Mesh:noexp] OR "kidney diseases"[tiab]))

Additional file 1: Appendix 2: NOS Criteria

**Selection**

1) Representativeness of the exposed cohort

a) truly representative of the average in the community *****

b) somewhat representative of the average in the community *****

c) selected group of users eg nurses, volunteers

d) no description of the derivation of the cohort

2) Selection of the non exposed cohort

a) drawn from the same community as the exposed cohort *****

b) drawn from a different source

c) no description of the derivation of the non exposed cohort

3) Ascertainment of exposure

a) secure record (eg surgical records) *****

b) structured interview *****

c) written self report

d) no description

4) Demonstration that outcome of interest was not present at start of study

a) yes *****

b) no

**Comparability**

1) Comparability of cohorts on the basis of the design or analysis

a) study controls for age *****

b) study controls for any additional factor: Gender *****

**Outcome**

1) Assessment of outcome

a) independent blind assessment *****

b) record linkage *****

c) self report

d) no description

2) Was follow-up long enough for outcomes to occur

a) yes - >1 year *****

b) no

3) Adequacy of follow up of cohorts

a) complete follow up - all subjects accounted for *****

b) subjects lost to follow up unlikely to introduce bias - small number lost - > 80 % *****

c) follow up rate < 80% and no description of those lost

d) no statement

Additional file 1: Appendix 3: Publication Bias Funnel Plots
